# Evaluation of FASTinov for rapid antimicrobial susceptibility testing in *Pseudomonas aeruginosa*

**DOI:** 10.1038/s41598-025-12137-w

**Published:** 2025-08-05

**Authors:** Carmen Cintora-Mairal, José Manuel Ortiz de la Rosa, Cidália Pina-Vaz, Guillermo Martín-Gutiérrez, José Antonio Lepe

**Affiliations:** 1 Unidad de Enfermedades Infecciosas, Microbiología y Parasitología Clínica, Hospital Universitario Virgen del Rocío, Seville, Spain; 2 Instituto de Biomedicina de Sevilla (IBiS), Hospital Universitario Virgen del Rocío, CSIC/Universidad de Sevilla, Seville, Spain; 3 Centro de Investigación Biomédica en Red de Enfermedades Infecciosas (CIBERINFEC), Instituto de Salud Carlos III, Madrid, Spain; 4 RISE-Health, Department of Pathology, Faculty of Medicine, University of Porto, Porto, Portugal; 5 FASTinov, S.A, Porto, Portugal; 6 Department of Health Sciences, Loyola Andalucía University, Seville, Spain

**Keywords:** FASTinov, Flow cytometry, Rapid susceptibility testing, *Pseudomonas aeruginosa*, Infection, Molecular medicine

## Abstract

*Pseudomonas aeruginosa* is a critical priority pathogen due to its high resistance rates and limited treatment options. Rapid antimicrobial susceptibility testing (rAST) is essential to optimize therapy, particularly because resistance mechanisms in *P. aeruginosa* often arise from adaptive genetic expression rather than specific resistance genes. FASTinov, a flow cytometry-based rAST system, was developed to address this challenge by providing phenotypic susceptibility results within hours. In this study, we assessed FASTinov’s performance for rapid antimicrobial susceptibility testing (rAST) in 100 *P. aeruginosa* strains, using broth microdilution as the reference method. For clinical relevance, we also included the MicroScan WalkAway system as a comparator. FASTinov demonstrated a categorical agreement of 99% across the tested antibiotics, with particularly strong agreement for cephalosporins. Notably, FASTinov significantly reduced the time to results, delivering susceptibility data in an average of 3.15 h (CI 95% 3.13–3.17), compared to 28.88 h (CI 95% 20.71–37.04) for standard methods. Additionally, the system exhibited lower rates of major and very major errors compared to MicroScan, further reinforcing its reliability. These findings highlight FASTinov as a promising rAST system capable of providing rapid and accurate susceptibility profiles directly from bacterial colonies. Further research is warranted to evaluate its clinical impact on patient outcomes and cost-effectiveness.

**Supplementary Information:**

The online version contains supplementary material available at 10.1038/s41598-025-12137-w.

## Introduction

*Pseudomonas aeruginosa* is a major opportunistic pathogen in both community and healthcare settings^1^. Infections caused by multidrug-resistant (MDR) *P. aeruginosa* strains are associated with increased mortality, prolonged hospital stays, and higher healthcare costs^2,3^. A key challenge in their management is the limited predictive value of molecular diagnostic methods, since resistance frequently arises from adaptive changes—such as AmpC overexpression, porin loss, or efflux pump activation—that are not detectable by genotypic assays^4^. This highlights the need for rapid phenotypic antimicrobial susceptibility testing (AST) methods capable of detecting functional resistance mechanisms in real time.

Among rapid AST commercially available systems, Accelerate Pheno provides phenotypic susceptibility results in approximately seven hours^5^, while the QMAC-dRAST System reports a total turnaround time of six to seven hours^6^. Additional technologies, including fluorescence-based assays, typically yield results within four to six hours^7^. Although these platforms represent a clear advancement over conventional methodologies, most remain dependent on bacterial growth and have been primarily validated using positive blood cultures. Importantly, their performance in *P. aeruginosa* remains insufficiently characterized, particularly when susceptibility testing is performed directly from bacterial colonies.

FASTinov (Porto, Portugal) has recently developed an innovative AST method based on flow cytometry. This system was designed to address the need for faster phenotypic resistance detection by enabling a growth-independent assessment of bacterial cell damage after exposure to antibiotics. The test is performed directly from isolated bacterial colonies and provides susceptibility results within 2–3 h, significantly reducing turnaround time compared to conventional methods. Previous studies have reported high sensitivity and specificity when using this system directly from positive blood cultures^8,9^. Its application for antimicrobial susceptibility testing in bacterial colonies, although described on instructions for use, remains insufficiently explored. This study aims to evaluate the performance of the FASTinov system for rapid antimicrobial susceptibility testing (rAST) from *P. aeruginosa* colonies, focusing on its accuracy in comparison to standard reference methods and the time required to obtain diagnostic results, with the goal of advancing its clinical applicability.

## Results

**Fig. 1:**
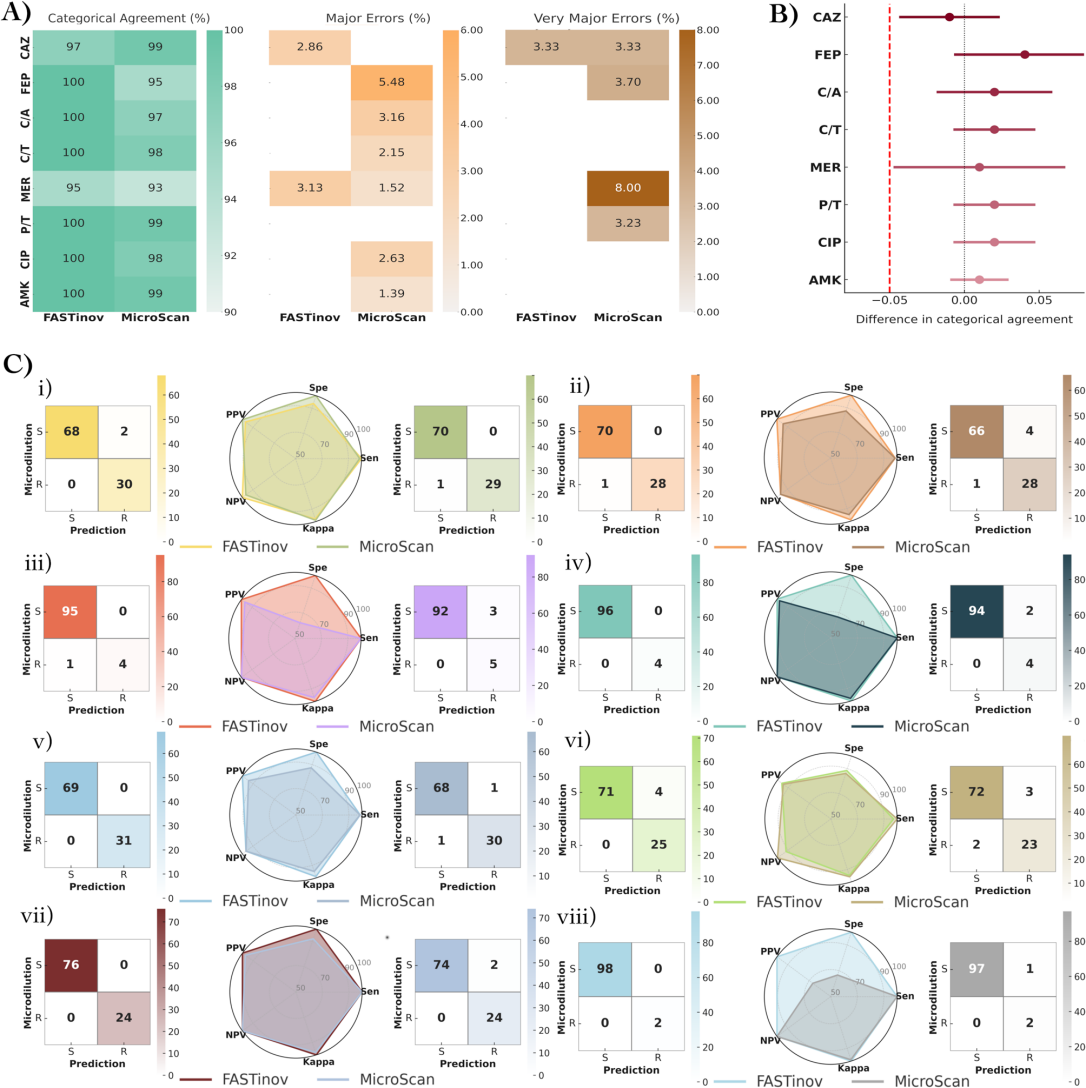
**(A)** Heatmaps display the performance of FASTinov and MicroScan for eight antibiotics, showing categorical agreement (CA), major errors (ME), and very major errors (VME) compared to the broth microdilution reference method. Color intensity corresponds to the percentage value, with harmonized scales across panels to facilitate visual comparison. Numeric values within each cell represent the percentage for each metric and technique. **(B)** Non-inferiority analysis comparing FASTinov and MicroScan. Difference in categorical agreement between FASTinov and MicroScan is shown for each antibiotic, with 95% confidence intervals. A non-inferiority margin of − 5% (red dashed line) was predefined. A positive value indicates higher agreement for FASTinov. **(C)** Comparative performance of FASTinov and MicroScan. Each panel includes two confusion matrices (left: FASTinov; right: MicroScan) and a central radar chart summarizing diagnostic performance metrics: sensitivity (Sen), specificity (Sep), positive predictive value (PPV), negative predictive value (NPV), and Cohen’s kappa coefficient (Kappa). The eight antibiotics evaluated are: (i) CAZ (ceftazidime), (ii) FEP (cefepime), (iii) C/A (ceftazidime–avibactam), (iv) C/T (ceftolozane–tazobactam), (v) MER (meropenem), (vi) P/T (piperacillin–tazobactam), (vii) CIP (ciprofloxacin), and (viii) AMK (amikacin).

The performance of the FASTinov system was evaluated on 100 *P. aeruginosa* strains and compared to the reference broth microdilution method. In addition, the MicroScan WalkAway system was included as a comparator, as it is the routine method used in our hospital for susceptibility testing. Overall, FASTinov demonstrated high reliability, achieving an average categorical agreement (CA) of 99% across all tested antibiotics. The highest CA (100%) was observed for cefepime, piperacillin/tazobactam, ceftolozane/tazobactam, ceftazidime/avibactam, ciprofloxacin, and amikacin, with no major errors (ME) or very ME (VME) (Fig. 1A). However, meropenem and ceftazidime showed lower CA values of 95% and 97%, respectively. Meropenem displayed the highest discrepancy rate, primarily due to minor errors (3%), while ceftazidime exhibited 2.86% ME and 3.33% VME, indicating potential misclassification of resistant strains as susceptible, which could carry significant clinical implications. Similarly, MicroScan results were compared to the reference method, showing an overall CA of 97%. The highest CA (99%) was observed for ceftazidime, amikacin, and piperacillin/tazobactam, while meropenem had the lowest CA (93%), followed by cefepime (95%). Minor errors were most frequent with meropenem (4%), while major errors were observed for most antibiotics except ceftazidime and piperacillin/tazobactam. Very major errors were highest for meropenem (8%), followed by cefepime (3.7%) and ceftazidime (3.33%), raising concerns regarding the potential underestimation of resistance (Fig. 1A, Supplementary Table 1). To evaluate the diagnostic performance of FASTinov compared to MicroScan, we conducted a non-inferiority analysis using categorical agreement as the primary outcome measure (Fig. 1B). The difference in agreement was calculated for each antibiotic, along with 95% confidence intervals. A non-inferiority margin of − 5% was predefined. FASTinov demonstrated non-inferiority to MicroScan for all eight antibiotics tested. For most antibiotics, the difference in agreement favored FASTinov, with confidence intervals entirely above the − 5% margin. These findings highlight the strong performance of FASTinov for *P. aeruginosa*, with fewer discrepancies compared to MicroScan.

In Fig. 1C, we can observe that FASTinov demonstrated consistently high sensitivity (≥ 97.3%) across all tested antibiotics in comparison with MicroScan, with perfect specificity (100%) for most agents except meropenem (88.5%), achieving overall high accuracy (≥ 98%) and strong agreement metrics, reflected in Youden index values reaching 100% for several antibiotics (Supplementary Table 2). The kappa coefficient further supported FASTinov’s reliability, exceeding 95 for most antibiotics, indicating almost perfect agreement with the reference method. In comparison, MicroScan exhibited similarly high sensitivity (≥ 98.5%), but specificity varied considerably, particularly for ceftolozane/tazobactam (66.7%) and ceftazidime/avibactam (62.5%), leading to lower Youden index values (62.5-66.7%) and reduced kappa coefficients (75.4–79.5) for these agents, suggesting moderate agreement (Supplementary Table 2). Despite both systems demonstrating strong overall performance, FASTinov achieved greater specificity, categorical agreement, and kappa values, highlighting its potential advantage in clinical applications where accurate resistance classification is crucial.

### Time to results

The time required to obtain antimicrobial susceptibility testing results using FASTinov was estimated and compared with the standard routine workflow in our laboratory. The comparison was performed for 51 samples and revealed a significant reduction in the time required for FASTinov results compared to the routine workflow, which utilizes the MicroScan system. On average, FASTinov provided results in just 3.15 h (CI 95% 3.13–3.17), compared with the routine workflow that required an average of 28.88 h (CI 95% 20.71–37.04).

## Discussion

Rapid and accurate antimicrobial susceptibility testing is essential for optimizing treatment strategies, particularly for *P. aeruginosa*. Genotypic methods are limited to detecting known resistance genes and cannot provide a comprehensive susceptibility profile, especially for *P. aeruginosa*. FASTinov, a flow cytometry-based system, was developed to address this gap by enabling rapid phenotypic resistance detection, not growth-dependent, directly from bacterial colonies. A multiparametric cell analysis including size, complexity and fluorescence of treated cells are compared with non-treated cells with a proprietary software. When performed from colonies bacteria need to grow in a broth for a short period in order to be in the exponential growth phase essential for most drug activity. In this study, FASTinov demonstrated superior categorical agreement (99%) compared to MicroScan (97%) and achieved superior specificity and kappa values, particularly for cephalosporins. Additionally, FASTinov met the FDA-recommended performance criteria, with very major errors (VME) below 1%, providing results in just 3 h, compared to the 18–24 h required for automated reading by the MicroScan system, plus the time needed to review the panels and generate the report in the patient’s medical record.

Recent literature emphasizes the critical role of rapid antimicrobial susceptibility testing in optimizing the treatment of infections caused by *P. aeruginosa*. Rates of inadequate therapy for these infections exhibit considerable variability, ranging from 24 to 80% depending on the clinical setting and patient population. For instance, Eklöf et al.^10^. reported that 80% of patients with pulmonary *P. aeruginosa* infections received inappropriate therapy, while Cillóniz et al.^11^. documented a rate of 64% for community-acquired pneumonia cases caused by this pathogen, rising to 77% in infections involving multidrug-resistant strains. Martinez-Nadal et al.^12^. observed that 24% of high-risk neutropenic patients with bacteremia caused by *P. aeruginosa* received inappropriate empirical therapy. Moreover, inadequate therapy has been also linked to extended hospital stays and higher healthcare costs^13,14^.

Given these findings, the implementation of rapid susceptibility testing emerges as a critical priority. However, most studies evaluating rAST methods include multiple bacterial species, often leading to limited *P. aeruginosa* sample sizes. Additionally, many studies focus solely on direct positive blood culture testing, whereas our study represents a unique approach by evaluating a rAST method directly from culture-grown colonies. Thus, the study performed by Pancholi et al.^15^. demonstrated that the Accelerate Pheno SYSTEM provides results within 5 h with a CA of ≥ 90% for 21 strains of *P. aeruginosa*, but with VME ≥ 5% for multiple antibiotics, including ceftazidime, piperacillin-tazobactam or amikacin. A more recent study by Sikorski et al.^16^. evaluated the performance of the Accelerate PhenoTest BC kit against a collection of 144 *P. aeruginosa* isolates, reporting a CA of ≥ 85%, with a VME of 7.9% for ceftazidime and minor errors of 10.4% for meropenem, indicating challenges in accurately detecting resistance for certain antibiotics. Other study evaluating the QMAC-dRAST system (QuantaMatrix) demonstrated a 92.9% concordance with the standard disk diffusion method for Gram-negative blood culture isolates, but among the 100 strains analyzed, only 9 were *P. aeruginosa*, limiting its applicability^17^. A recent study by Couchot et al.^18^. evaluated the Reveal rapid AST system using 200 *P. aeruginosa* strains, reporting a CA of 96.1% and very major errors (VME) of 1.6%, providing results within an average time of 6 h and 22 min. Building on these findings, our study demonstrated that the system achieves a higher CA than previously reported rAST methods, with superior performance across multiple antibiotics. In addition, given its ability to deliver results in just 3 h and its high agreement with microdilution, FASTinov presents itself as a promising clinical tool for rapid antimicrobial susceptibility testing, potentially improving early therapeutic decision-making and antimicrobial stewardship efforts. However, a potential limitation of the method is the need for strict adherence to incubation times and timely processing at each step, which requires the continuous involvement of a laboratory technician to ensure accuracy and reproducibility of the results.

In conclusion, our study showed the FASTinov system as a promising tool for guiding early and targeted antimicrobial therapy for *P. aeruginosa* infections. Compared to MicroScan, FASTinov demonstrated higher accuracy, faster turnaround times, and fewer errors. Its ability to perform susceptibility testing directly from bacterial colonies represents a significant advancement, expanding its applicability to a wider range of clinical samples and enhancing its utility, particularly in severe infections. However, further studies are needed to assess the real-world clinical impact of FASTinov, specifically its role in optimizing targeted therapy and improving patient outcomes. Additionally, as rapid AST systems typically incur higher costs than conventional susceptibility testing methods, a thorough cost-effectiveness analysis will be crucial to determining its feasibility for routine implementation in clinical microbiology laboratories.

## Materials and methods

### Bacterial strains

A total of 100 *P. aeruginosa* strains were included in the study, consisting of 99 clinical isolates collected from samples processed at the Microbiology Laboratory of Virgen del Rocío University Hospital (Seville, Spain) between 2021 and 2024, and one reference strain (*P. aeruginosa* ATCC 27853). All clinical strains were identified using MALDI-TOF mass spectrometry (Bruker, Germany).

### Antimicrobial susceptibility testing

**Fig. 2:**
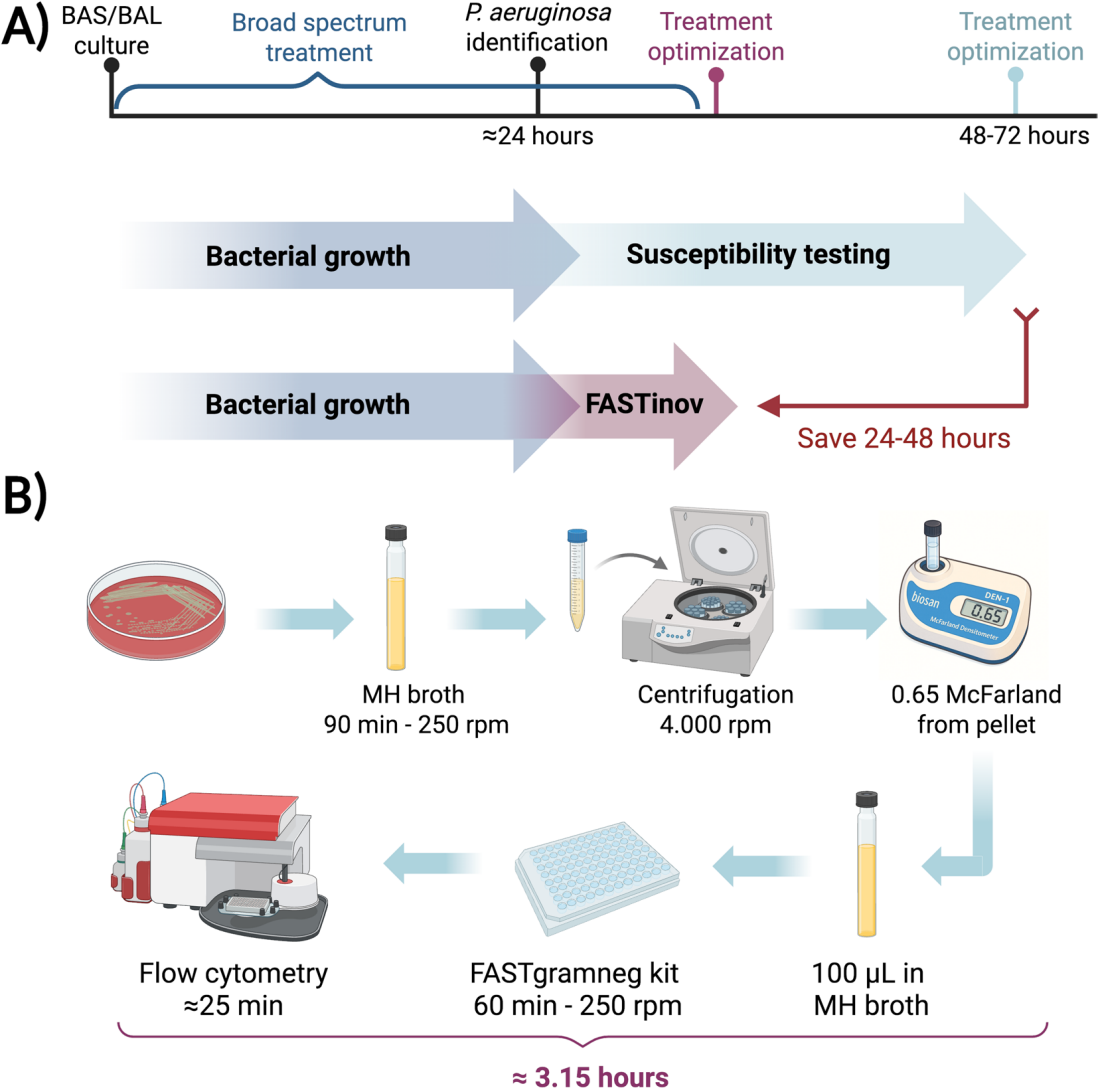
**(A)** Schematic comparison of the conventional antimicrobial susceptibility testing (AST) workflow and the FASTinov-based rapid AST (rAST) workflow for *Pseudomonas aeruginosa* infections. The conventional workflow requires overnight incubation for AST results, whereas the FASTinov rAST workflow performs susceptibility testing directly from isolated colonies, significantly reducing the turnaround time. **(B)** Overview of the FASTinov rAST workflow, showing the main steps from colony preparation to fluorescence-based detection of antimicrobial susceptibility. Original figure created with BioRender.com.

Antimicrobial susceptibility testing was determined by three different methods: (i) Flow Cytometry Assay (FASTinov): A novel flow cytometry-based method was also evaluated with the aim of reducing the time needed to obtain an antibiotic sensitivity report (Fig. 2A). FASTinov is a growth-independent AST method in which each well of the panel contains a specific antimicrobial combined with selected fluorescent probes that target early cellular damage. After a short incubation period, susceptible bacteria exhibit structural lesions, leading to altered fluorescence signals, whereas resistant bacteria maintain their integrity, showing no significant change. These differential patterns allow for rapid interpretation of susceptibility profiles without relying on bacterial replication or growth (Supplementary Fig. 1). To apply this principle in our experimental workflow, 2–3 colonies of *P. aeruginosa* were selected and incubated in 7 mL of MHB for 1.5 h at 37 °C with shaking at 250 rpm. The samples were then centrifuged (5.000 rpm 5 min), the supernatant was discarded, and the pellet was resuspended in 1 mL of saline to achieve a suspension adjusted to 0.65 McFarland. From this suspension, 100 µL were transferred to 7 mL of MHB and used to inoculate GRAMNEGcryo1 panels by adding 100 µL to each well. The entire preparation process, from centrifugation to panel inoculation, takes approximately 10–15 min. The panels were incubated at 37 °C for 1 h with shaking at 250 rpm. Following incubation, flow cytometric analysis was performed, (Fig. 2B), a process that requires approximately 25 min. (ii) MicroScan WalkAway system: Susceptibility profiles for all strains were initially determined using the NMR1 panel on the MicroScan WalkAway system (Beckman Coulter, USA), the routine method employed in our hospitals for antimicrobial susceptibility testing. (iii) Broth Microdilution: Minimum inhibitory concentrations (MICs) were assessed by the broth microdilution method using Mueller-Hinton broth (MHB), following the guidelines established by the European Committee on Antimicrobial Susceptibility Testing (EUCAST) (2024, Breakpoint tables for interpretation of MICs and zone diameters, http://www.eucast.org*).* All MIC values for the included isolates are provided in Supplementary Table 3.

The FASTinov panels are proprietary dried 96-well microplate format with the antimicrobials spanning breakpoint concentrations according to EUCAST criteria and a fluorescent dye: 2 controls (C1-non-treated cells and C2- dead cells), ceftazidime 8 µg/ml, cefepime 8 µg/ml, piperacillin/tazobactam 16/4 µg/ml, meropenem 2 and 8 µg/ml, ceftolozane/tazobactam 4/4 µg/ml, ceftazidime/avibactam 8/4 µg/ml, ciprofloxacin 0.5 µg/ml and amikacin 16 µg/ml.

#### Data analysis

Interpretation of flow cytometry data was performed using BioFASTast software, developed by FASTinov. A multiparametric analysis is performed comparing size (SSC), complexity (FSC) and intensity of fluorescence of the treated cells with non-treated cells (C1) (Supplementary Fig. 2). Cut-off values were defined to classify the susceptibility of the strains based on the analysis of a lot of well characterized strains. The program reports whether the strain is “susceptible” (S), “susceptible to increased doses” (I; EUCAST), “intermediate” (I; CLSI) or “resistant” (R). Another control (C2) with a killing agent is present in the panel to ensure that the fluorescent dyes perform as expected. To evaluate diagnostic testing accuracy, we assessed multiple parameters, including sensitivity, specificity, positive predictive value (PPV), negative predictive value (NPV), and categorical agreement (CA) between the results of FASTinov, MicroScan and broth microdilution methods^19^. In addition, data from the laboratory information system (LIS) were used to determine the time elapsed from the identification of *P. aeruginosa* by MALDI-TOF to the reporting and availability of antimicrobial susceptibility results in the patient’s medical record.

To assess the comparative performance of FASTinov and MicroScan against the reference standard (broth microdilution), we conducted a non-inferiority analysis for each antibiotic tested. Susceptibility results were categorized as “Susceptible” or “Resistant”, considering both “S” and “I” as susceptible. The categorical agreement between each method and microdilution was calculated. The difference in agreement (FASTinov minus MicroScan) was computed along with the corresponding 95% confidence interval (CI) using the Wald method for two independent proportions. A non-inferiority margin (Δ) of − 5% was pre-specified.

## Electronic supplementary material

Below is the link to the electronic supplementary material.

Supplementary Material 1

## Data Availability

The datasets generated and analyzed during the current study are available from the corresponding author upon reasonable request. Due to ethical and privacy considerations, some data may be subject to access restrictions. All relevant processed data supporting the findings of this study are included in the manuscript.
