# Supplementary material for: Evaluation of FASTinov for rapid antimicrobial susceptibility testing in Pseudomonas aeruginosa
Source: Sci Rep. 2025 Aug 5;15:28649. doi: 10.1038/s41598-025-12137-w (PMC12325984; doi:10.1038/s41598-025-12137-w)
Supplement: Supplementary file 1 — Supplementary Material 1 [file 41598_2025_12137_MOESM1_ESM.docx]

**Supplemental material**

Carmen Cintora-Mairal, José Manuel Ortiz de la Rosa, Cidália Pina-Vaz , Guillermo Martín-Gutiérrez^1, 2, 3,6^, José Antonio Lepe ^1, 2, 3^.

This supplemental material has been provided by the authors to give readers additional information about their work. Contents are presented in the order that appeared in the text.

| **Table 1.** Overall summary of CA, mE, ME and VME rates in FASTinov and MicroScan compared with broth microdilution. | | | | | | | |  |
| --- | --- | --- | --- | --- | --- | --- | --- | --- |
|  |  |  |  |  |  |  |  |  |
| **A. FASTinov** | | | | | | | |  |
| **Antibiotic tested** | **S (N)** | **I (N)** | **R (N)** | **CA (%)** | **mE (%)** | **ME (%)** | **VME (%)** |  |
| **CAZ** | NA | 70 | 30 | 97 | - | 2,86 | 3,33 |  |
| **FEP** | NA | 73 | 27 | 100 | - | - | - |  |
| **P/T** | NA | 69 | 31 | 100 | - | - | - |  |
| **C/T** | 96 | NA | 4 | 100 | - | - | - |  |
| **C/A** | 95 | NA | 5 | 100 | - | - | - |  |
| **MER** | 64 | 11 | 25 | 95 | 3,00 | 3,13 | - |  |
| **CIP** | NA | 76 | 24 | 100 | - | - | - |  |
| **AMK** | 98 | NA | 2 | 100 | - | - | - |  |
|  |  |  |  |  |  |  |  |  |
| **B. MicroScan** | | | | | | | |  |
| **Antibiotic tested** | **S (N)** | **I (N)** | **R (N)** | **CA (%)** | **mE (%)** | **ME (%)** | **VME (%)** |  |
| **CAZ** | NA | 70 | 30 | 99 | - | - | 3,33 |  |
| **FEP** | NA | 73 | 27 | 95 | - | 5,48 | 3,70 |  |
| **P/T** | NA | 69 | 31 | 99 | - | - | 3,23 |  |
| **C/T** | 96 | NA | 4 | 98 | - | 2,15 | - |  |
| **C/A** | 95 | NA | 5 | 97 | - | 3,16 | - |  |
| **MER** | 64 | 11 | 25 | 93 | 4,00 | 1,52 | 8,00 |  |
| **CIP** | NA | 76 | 24 | 98 | - | 2,63 | - |  |
| **AMK** | 98 | NA | 2 | 99 | - | 1,39 | - |  |
| S (susceptible); I (susceptible, increased exposure); R (resistant); CA (categorical agreement); mE (minor errors); ME (major errors); VME (very major errors); CAZ (ceftazidime); FEP (cefepime); P/T (piperacillin/tazobactam); C/T (ceftazidime/tazobactam); C/A (ceftazidime/avibactam); MER (meropenem); CIP (ciprofloxacin); AMK (amikacin). NA (Not Applicable). | | | | | | | | |

| **Table 2.** Diagnostic performance metrics of FASTinov and MicroScan compared to broth microdilution. Value (CI 95%). | | | | | | | | |  |
| --- | --- | --- | --- | --- | --- | --- | --- | --- | --- |
|  |  |  |  |  |  |  |  |  |  |
| **A. FASTinov** | | | | | | | | |  |
|  | **CAZ** | **FEP** | **P/T** | **C/T** | **C/A** | **MER** | **CIP** | **AMK** |  |
| **Sensitivity (%)** | 100 (92.1-100) | 98.6 (92.3-100) | 98.6 (92.3-100) | 100 (94.3-100) | 100 (94.3-100) | 97.3 (90.5-99.7) | 100 (02.9-100) | 100 (94.4-100) |  |
| **Specificity (%)** | 93.8 (79.2-99.2) | 100 (82.2-100) | 100 (82.2-100) | 100 (28.4-100) | 100 (35.9-100) | 88.5 (69.8-97.6) | 100 (79.6-100) | 100 (94.4-100) |  |
| **PPV (%)** | 97.1 (89.9-99.6) | 100 (92.3-100) | 100 (92.3-100) | 100 (94.3-100) | 100 (94.3-100) | 95.9 (88.6-99.2) | 100 (92.9-100) | 100 (94.4-100) |  |
| **NPV (%)** | 100 (83.3-100) | 96.6 (82.2-99.9) | 96.6 (82.2-99.9) | 100 (28.4-100) | 100 (35.9-100) | 92 (74-99) | 100 (79.6-100) | 100 (94.4-100) |  |
| **Accuracy (%)** | 98 (92.9-99.8) | 99 (94.4-100) | 99 (94.4-100) | 100 (94.6-100) | 100 (94.6-100) | 94.9 (88.6-98.3) | 100 (9.46-100) | 100 (94.6-100) |  |
| **Youden Index** | 93.8 (71.3-99.2) | 98.6 (74.5-100) | 98.6 (74-5-100) | 100 (22.7-100) | 100 (30.2-100) | 85.8 (60.3-97.3) | 100 (72.5-100) | 100 (3.8-100) |  |
| **Kappa** | 95.3 (88.8-100) | 97.5 (92.7-100) | 97.5 (92.7-100) | 100 (100) | 100 (100) | 89.8 (80.1-99.5) | 100 (100) | 100 (100) |  |
|  |  |  |  |  |  |  |  |  |  |
| **B. MicroScan** | | | | | | | | |  |
|  | **CAZ** | **FEP** | **P/T** | **C/T** | **C/A** | **MER** | **CIP** | **AMK** |  |
| **Sensitivity (%)** | 98.6 (92.3-100) | 98.5 (91.8-100) | 98.5 (91.8-100) | 100 (94.2-100) | 100 (94.1-100) | 100 (92.4-100) | 100 (92.7-100) | 100 (94.4-100) |  |
| **Specificity (%)** | 100 (82.8-100) | 87.5 (71-96.5) | 87.5 (71-96.5) | 66.7 (22.3-100) | 62.5 (24.5-91.5) | 86.2 (68.3-96.1) | 92.3 (74.9-99.1) | 66.7 (9.4-99.2) |  |
| **PPV (%)** | 100 (92.3-100) | 94.2 (85.8-98.4) | 94.2 (85.8-98.4) | 97.9 (92.6-99.7) | 96.8 (92-99.3) | 94.6 (86.7-98.5) | 97.3 (90.7-99.7) | 33.3 (0.8-90.6) |  |
| **NPV (%)** | 96.7 (82.8-99.9) | 96.6 (82.2-99.9) | 96.6 (82.2-99.9) | 100 (28.4-100) | 100 (35.9-100) | 100 (80.4-100) | 100 (79.6-100) | 100 (9.4-100) |  |
| **Accuracy (%)** | 99 (94.5-100) | 94.9 (88.5-98.3) | 94.9 (88.5-98.3) | 98 (92.9-99.8) | 97 (91.4-99.4) | 96 (90-98.9) | 98 (92.9-99.8) | 99 (94.5-100) |  |
| **Youden Index** | 98.6 (75.1-100) | 86 (62.8-96.5) | 86 (62.8-96.5) | 66.7 (16.5-95.7) | 62.5 (18.6-91.5) | 86.2 (60.7-96.1) | 92.3 (67.6-99.1) | 66.7 (3.8-99.2) |  |
| **Kappa** | 97.6 (92.8-100) | 88.1 (77.9-98.2) | 88.1 (77.9-98.2) | 79 (50.8-100) | 75.4 (48.8-100) | 86.8 (75.5-98) | 94.7 (87.3-100) | 79.5 (40.3-100) |  |
| PPV (positive predictive value); NPV (negative predictive value); kappa (CAZ (ceftazidime); FEP (cefepime); P/T (piperacillin/tazobactam); C/T (ceftazidime/tazobactam); C/A (ceftazidime/avibactam); MER (meropenem); CIP (ciprofloxacin); AMK (amikacin). | | | | | | | | | |


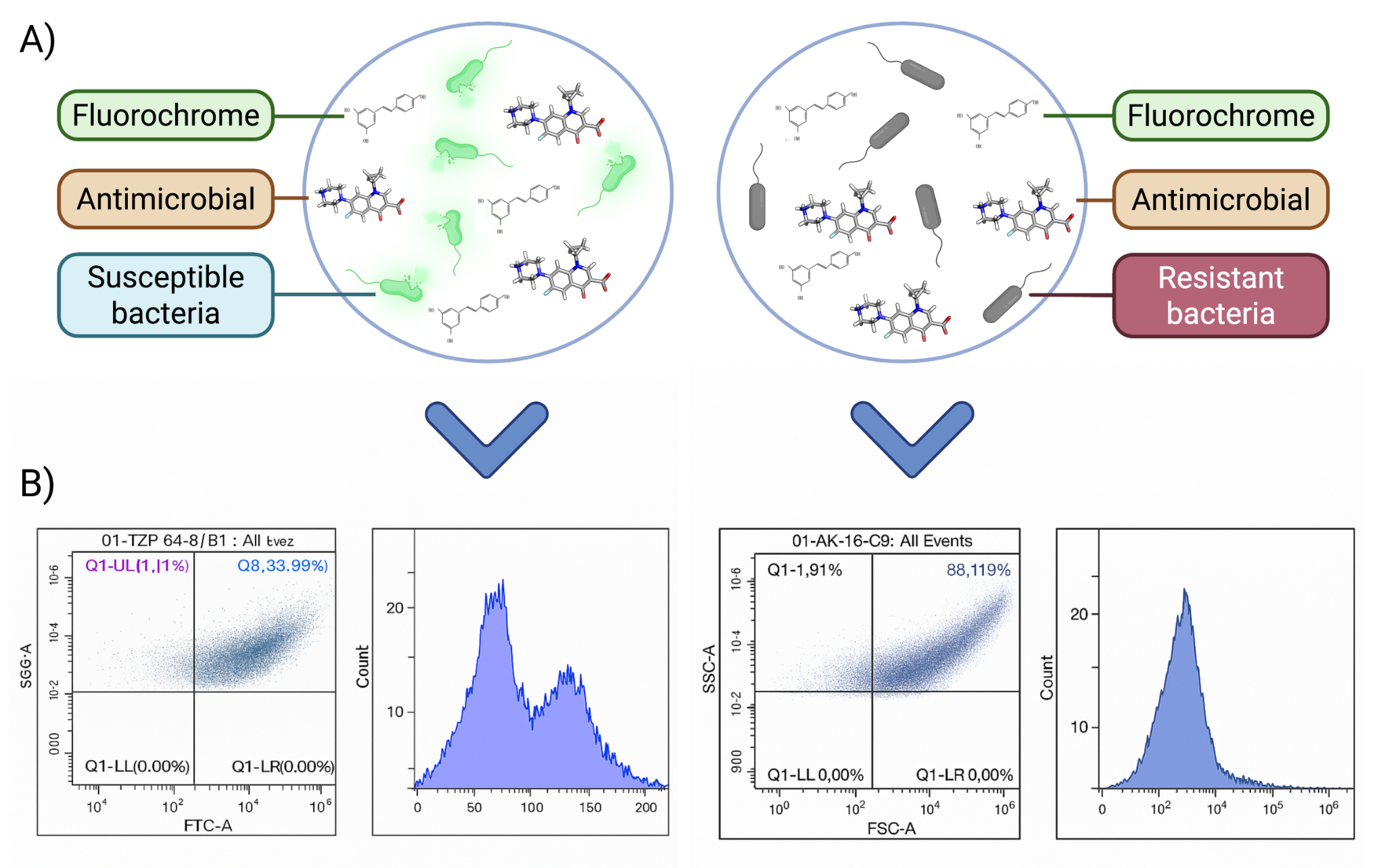


**Supplementary Figure 1.** **A)** Schematic representation of the FASTinov method. Each panel well combines an antimicrobial and a fluorescent probe. After 60 minutes of incubation, susceptible bacteria exhibit fluorescence alterations due to cellular damage, while resistant strains remain unaffected. **B)** Following the incubation step, flow cytometry analysis is performed. This panel shows a representative scattergram and fluorescence histograms illustrating the differential profiles of a bacterial strain susceptible and resistant to a given antibiotic. The observed differences in fluorescence intensity and light-scattering properties reflect membrane integrity and viability status, enabling rapid discrimination of antimicrobial susceptibility. Original figure created with [BioRender.com](https://www.biorender.com/).

| **Table 3.** Minimum Inhibitory Concentrations (MICs) of antibiotics tested against 100 *P. aeruginosa* strains. | | | | | | | | | | | | | | | | |
| --- | --- | --- | --- | --- | --- | --- | --- | --- | --- | --- | --- | --- | --- | --- | --- | --- |
| **Strain** | **Amikacin** | | **Ciprofloxacin** | | **Ceftazidime-**  **Avibactam** | | **Ceftolozane-**  **Tazobactam** | | **Piperacillin- Tazobactam** | | **Cefepime** | | **Ceftazidime** | | **Meropenem** | |
|  | **MIC/Category** | | **MIC/Category** | | **MIC/Category** | | **MIC/Category** | | **MIC/Category** | | **MIC/Category** | | **MIC/Category** | | **MIC/Category** | |
| **ATCC**  **27853** | 2 | S | 0.125 | I | 0.5/4 | S | 0.25 | S | 8/4 | I | 0.5 | I | 1 | I | 0.25 | S |
| **2** | 8 | S | 4 | R | <2/4 | S | 4/4 | S | >256/4 | R | 16 | R | 64 | R | 17 | R |
| **3** | 2 | S | 2 | R | 8/4 | S | 2/4 | S | >128/4 | R | 32 | R | 16 | R | >64 | R |
| **4** | 2 | S | 0.5 | I | 0.5/4 | S | 0.5/4 | S | 4/4 | I | 16 | R | 1 | I | 32 | R |
| **5** | 4 | S | 0.06 | I | 1/4 | S | 32/4 | R | 8/4 | I | 16 | R | 16 | R | 2 | S |
| **6** | 6 | R | 0.26 | I | >64/4 | R | >32/4 | R | 16/4 | I | 32 | R | >64 | R | 16 | R |
| **7** | 2 | S | 2 | R | 8/4 | s | 4/4 | S | 4/4 | I | 32 | R | 16 | R | 32 | R |
| **8** | 2 | S | 0.5 | I | 4/4 | S | >32/4 | R | 16/4 | S | 16 | R | 16 | R | 16 | R |
| **9** | 16 | S | 0.25 | I | 4/4 | S | 0.25 | S | 16/4 | I | 16 | R | 16 | R | 16 | R |
| **10** | 1 | S | 0.125 | I | 8/4 | S | 0.25 | S | 16/4 | I | 8 | I | 8 | I | 32 | R |
| **11** | 2 | S | 0.5 | I | 1/4 | S | 0.5/4 | S | 4/4 | I | 8 | I | 4 | I | 8 | I |
| **12** | 8 | S | >16 | R | 4/4 | R | 32/4 | R | 16/4 | I | 128 | R | >64 | R | >64 | R |
| **13** | 1 | S | 0.125 | I | 2 | S | >64/4 | R | >256/4 | R | >128 | R | 128 | R | 16 | R |
| **14** | 8 | S | 1 | R | >64/4 | R | 8/4 | R | >256/4 | R | >128 | R | >128 | R | 0.5 | S |
| **15** | 8 | S | 16 | R | >64/4 | R | >64/4 | R | 16/4 | I | 64 | R | >128 | R | 32 | R |
| **16** | 2 | S | 2 | R | 0.5/4 | S | 4/4 | S | >256/4 | R | 32 | R | 64 | R | 8 | I |
| **17** | 4 | S | 16 | R | 8/4 | S | 4/4 | S | >256/4 | R | 32 | R | 32 | R | 64 | R |
| **18** | 2 | S | 0.5 | I | 0.5/4 | S | 2/4 | S | 4/4 | I | 1 | I | 8 | I | 2 | S |
| **19** | 0.5 | S | 2 | R | 4/4 | S | 0.25 | S | 16/4 | I | 8 | I | 4 | I | 16 | R |
| **20** | 4 | S | 0.25 | I | 1/4 | S | 0.5/4 | S | 16/4 | I | 2 | I | 1 | I | 8 | I |
| **21** | 4 | S | 0.25 | I | 2/4 | S | 4/4 | S | >128/4 | R | 2 | I | 32 | R | 8 | I |
| **22** | 16 | S | 2 | R | 1/4 | S | 0.25 | S | 16/4 | I | 4 | I | 2 | I | 2 | S |
| **23** | 32 | R | 0.125 | I | 1/4 | S | 0.25 | S | 16/4 | I | 16 | R | 32 | R | 16 | R |
| **24** | 2 | S | 1 | R | 2/4 | S | 4/4 | S | >256/4 | R | 16 | R | 65 | R | 16 | R |
| **25** | 2 | S | 1 | R | 8/4 | S | 0.25 | S | 4/4 | I | 16 | R | 8 | I | 8 | I |
| **26** | 16 | S | 0.125 | I | 1/4 | S | 2/4 | S | >256/4 | R | 16 | R | 128 | R | 16 | R |
| **27** | 4 | S | 2 | R | 1/4 | S | 2/4 | S | 8/4 | I | 8 | I | 2 | I | 16 | R |
| **28** | 4 | S | 0.5 | I | 0.5 | S | 0.5/4 | S | 4/4 | I | 32 | R | 128 | R | 8 | I |
| **29** | 2 | S | 0.25 | I | 0.5 | S | 4/4 | S | >256/4 | R | 16 | R | 128 | R | 32 | R |
| **30** | 4 | S | 0.125 | I | 0.5 | S | 0.25 | S | 128/4 | R | 16 | R | 32 | R | 4 | I |
| **31** | 0.125 | S | 0.125 | I | 1/4 | S | 0.25 | S | 16/4 | I | 8 | I | 4 | I | 8 | I |
| **32** | 4 | S | 0.06 | I | 1/4 | S | 4/4 | S | >256/4 | R | 16 | R | 128 | R | 16 | R |
| **33** | 1 | S | 0.25 | I | 2/4 | S | 0.5/4 | S | 16/4 | I | 1 | I | 2 | I | 0.5 | s |
| **34** | 4 | S | 0.25 | I | 1/4 | S | 0.25 | S | 16/4 | I | 8 | I | 8 | I | 4 | I |
| **35** | 8 | S | 0.125 | I | 1/4 | S | 2/4 | S | >256/4 | R | 16 | R | 32 | R | 16 | R |
| **36** | 4 | S | 0.5 | I | 2/4 | S | 0.25 | S | 16/4 | I | 2 | I | 2 | I | 8 | I |
| **37** | 4 | S | 0.25 | I | 0.5 | S | 0.5/4 | S | 8/4 | I | 2 | I | 4 | I | 8 | I |
| **38** | 4 | S | 0.06 | I | 0.5 | S | 0.25 | S | >256/4 | R | 16 | R | 64 | R | 16 | R |
| **39** | 4 | S | 8 | R | 0.25/4 | S | 0.5/4 | S | 1/4 | I | 2 | I | 1 | I | 0.25 | S |
| **40** | 8 | S | 2 | R | 0.25/4 | s | 0.25 | S | 2/4 | I | 4 | I | 2 | I | 2 | S |
| **41** | 8 | S | 0.5 | I | 2/4 | S | 2/4 | S | 256/4 | R | 8 | I | 8 | I | 4 | I |
| **42** | 2 | S | 0.125 | I | 2/4 | S | 0.25 | S | >256/4 | R | 16 | R | >64 | R | 8 | I |
| **43** | 2 | S | 0.03 | I | 2/4 | S | 0.25 | S | 4/4 | I | 0.25 | I | 1 | I | 2 | S |
| **44** | 4 | S | 16 | R | 2/4 | S | 0.25 | S | 8/4 | S | 8 | I | 1 | I | 1 | S |
| **45** | 2 | S | 0.06 | I | 2/4 | S | 0.5/4 | S | 16/4 | I | 1 | I | 2 | I | 4 | I |
| **46** | 2 | S | 0.5 | I | 8/4 | S | 4/4 | S | >256/4 | R | 64 | R | >128 | R | 16 | R |
| **47** | 4 | S | 0.25 | I | 1/4 | S | 0.5/4 | S | 16/4 | I | 2 | I | 4 | I | 8 | I |
| **48** | 4 | S | 0.5 | I | 1/4 | S | 0.25 | S | 16/4 | I | 8 | I | 8 | I | 16 | R |
| **49** | 8 | S | >16 | R | 1/4 | S | 0.25 | S | 4/4 | I | 16 | R | 8 | I | 16 | R |
| **50** | 2 | S | 0.06 | I | 0.5/4 | S | 0.25/4 | s | 8/4 | I | 0.5 | I | 2 | I | 0.5 | S |
| **51** | 2 | S | 0.06 | I | 0.5/4 | S | 1/4 | S | 2/4 | I | 1 | I | 2 | I | 0.125 | S |
| **52** | 2 | S | 0.06 | I | 0.5/4 | S | 0.5/4 | S | 2/4 | I | 1 | I | 2 | I | 0.125 | S |
| **53** | 4 | S | 0.125 | I | 0.5/4 | S | 0.25/4 | S | 2/4 | I | 2 | I | 0.5 | I | 0.125 | S |
| **54** | 4 | S | 0.06 | I | 0.5/4 | S | 0.5/4 | S | 4/4 | I | 1 | I | 2 | I | 0.125 | S |
| **55** | 4 | S | 0.125 | I | 1/4 | S | 0.5/4 | S | 4/4 | I | 2 | I | 1 | I | 0.125 | S |
| **56** | 4 | S | 0.25 | I | 1/4 | S | 0.5/4 | S | 16/4 | I | 4 | S | 8 | I | 2 | S |
| **57** | 8 | S | 0.06 | I | 1/4 | S | 0.5/4 | S | 16/4 | I | 1 | I | 2 | I | 1 | S |
| **58** | 4 | S | 0.125 | I | 1/4 | S | 0.5/4 | S | 16/4 | I | 2 | I | 2 | I | 0.5 | S |
| **59** | 2 | S | 0.125 | I | 1/4 | S | 0.5/4 | S | 8/4 | I | 1 | I | 1 | I | 2 | S |
| **60** | 8 | S | 0.25 | I | 1/4 | S | 0.5/4 | S | 16/4 | I | 2 | I | 2 | I | 2 | S |
| **61** | 1 | S | 0.6 | I | 1/4 | S | 0.5/4 | S | 16/4 | I | 1 | I | 2 | I | 0.25 | S |
| **62** | 2 | S | 0.5 | I | 2/4 | S | 0.5/4 | S | 8/4 | I | 1 | I | 2 | I | 0.25 | S |
| **63** | 4 | S | 0.25 | I | 1/4 | S | 0.25 | S | 16/4 | I | 2 | I | 4 | I | 0.25 | S |
| **64** | 4 | S | 0.06 | I | 1/4 | S | 0.5/4 | S | 16/4 | I | 2 | I | 4 | I | 1 | S |
| **65** | 8 | S | 0.06 | I | 1/4 | S | 0.25 | S | 4/4 | I | 2 | I | 4 | I | 0.5 | S |
| **66** | 4 | S | 0.125 | I | 1/4 | S | 0.25 | S | 16/4 | I | 1 | I | 1 | I | 1 | S |
| **67** | 2 | S | 0.25 | I | 2/4 | S | 0.5/4 | S | 16/4 | I | 4 | I | 1 | I | <0.125 | S |
| **68** | 1 | S | 0.25 | I | 4/4 | S | 0.5/4 | S | 16/4 | I | 1 | I | 2 | I | 0.25 | S |
| **69** | 2 | S | 0.25 | I | 2/4 | S | 0.5/4 | S | 16/4 | I | 4 | I | 2 | I | 0.25 | S |
| **70** | 4 | S | 0.06 | I | 0.5 | S | 0.5/4 | S | 4/4 | I | 4 | I | 2 | I | 0.125 | S |
| **71** | 2 | S | 0.5 | I | 4/4 | S | 0.25 | S | 128/4 | I | 8 | I | 8 | I | 0.25 | S |
| **72** | 2 | s | 0.5 | I | 2/4 | S | 0.25 | S | 16/4 | I | 2 | I | 2 | I | 0.125 | S |
| **73** | 2 | S | 0.5 | I | 0.5 | S | 0.5/4 | S | 16/4 | I | 4 | I | 0.5 | I | <0.125 | S |
| **74** | 1 | S | 0.5 | I | 0.5 | S | 0.5/4 | S | 4/4 | I | 4 | I | 4 | I | <0.125 | S |
| **75** | 16 | S | 1 | R | >64/4 | R | >32/4 | R | >128/4 | R | 128 | R | >64 | R | >64 | R |
| **76** | 2 | S | 0.125 | I | 2/4 | S | 0.5/4 | S | 8/4 | I | 4 | S | 2 | I | 1 | S |
| **77** | 0.5 | S | 2 | R | 1/4 | S | 1/4 | S | 4/4 | I | 8 | I | 2 | I | <0.125 | S |
| **78** | 2 | S | 0.25 | I | 2/4 | S | 0.5/4 | S | 4/4 | I | 3 | I | 2 | I | 0.25 | S |
| **79** | 2 | S | 0.06 | I | 1/4 | S | 0.5/4 | S | 4/4 | I | 4 | I | 1 | I | 0.25 | S |
| **80** | 1 | S | 0.25 | I | 4/4 | S | 0.5/4 | S | 4/4 | I | 8 | I | 2 | I | 2 | S |
| **81** | 8 | S | 1 | R | 1/4 | S | 1/4 | S | 4/4 | I | 8 | I | 2 | I | 0.25/8 | S |
| **82** | 2 | S | 0.25 | I | 1/4 | S | 1/4 | S | 8/4 | I | 8 | I | 2 | I | 0.5 | S |
| **83** | 4 | S | 2 | R | 2/4 | S | 1/4 | S | 8/4 | I | 8 | I | 2 | I | <0.125 | S |
| **84** | 2 | S | 0.125 | I | 1/4 | S | 0.5/4 | S | 4/4 | I | 2 | S | 2 | I | 1 | S |
| **85** | 2 | S | 0.125 | I | 1/4 | S | 0.5/4 | S | 4/4 | I | 4 | s | 4 | I | 2 | S |
| **86** | 1 | S | 0.5 | I | 1/4 | S | 0.5/4 | S | 4/4 | I | 8 | I | 1 | I | <0.125 | S |
| **87** | 4 | S | 0.125 | I | 4/4 | S | 0.5/4 | S | 4/4 | I | 2 | I | 1 | I | 0.5 | S |
| **88** | 4 | S | 0.06 | I | 1/4 | S | 0.5/4 | S | 4/4 | I | 5 | I | 1 | I | 1 | S |
| **89** | 2 | S | 0.125 | I | 4/4 | S | 2/4 | S | 32/4 | R | 8 | I | 8 | I | 0.5 | S |
| **90** | 1 | S | 0.06 | I | 1/4 | S | 0.5/4 | S | 4/4 | I | 2 | I | 1 | I | 0.5 | S |
| **91** | 2 | S | 0.125 | I | 8/4 | S | 2/4 | S | 16/4 | I | 32 | R | 32 | R | 1 | S |
| **92** | 2 | S | 0.125 | I | 0.5/4 | S | 0.5/4 | S | 4/4 | I | 2 | I | 0.5 | I | 0.25 | S |
| **93** | 1 | S | 0.25 | I | 0.5/4 | S | 0.5/4 | S | 4/4 | I | 2 | I | 0.5 | I | 0.5 | S |
| **94** | 4 | S | 0.125 | I | 0.5/4 | S | 0.5/4 | S | 16/4 | I | 1 | I | 2 | I | 2 | S |
| **95** | 2 | S | 2 | R | 0.5/4 | S | 0.5/4 | S | 4/4 | S | 8 | I | 2 | I | 0.125 | S |
| **96** | 2 | S | 0.03 | I | 1/4 | S | 0.5/4 | S | 8/4 | I | 1 | I | 2 | I | <0.125 | S |
| **97** | 8 | S | 0.25 | I | 2/4 | S | 1/4 | S | 4/4 | I | 8 | I | 1 | I | 0.25 | S |
| **98** | 8 | S | 0.125 | I | 1/4 | S | 1/4 | S | 4/4 | I | 4 | I | 4 | I | 2 | S |
| **99** | 2 | S | 0.06 | I | 1/4 | S | 1/4 | S | >128/4 | R | 32 | R | 128 | R | 2 | S |
| **100** | 2 | S | 0.06 | I | 1/4 | S | 0.25/4 | S | 8/4 | I | 1 | I | 2 | I | <0.125 | S |


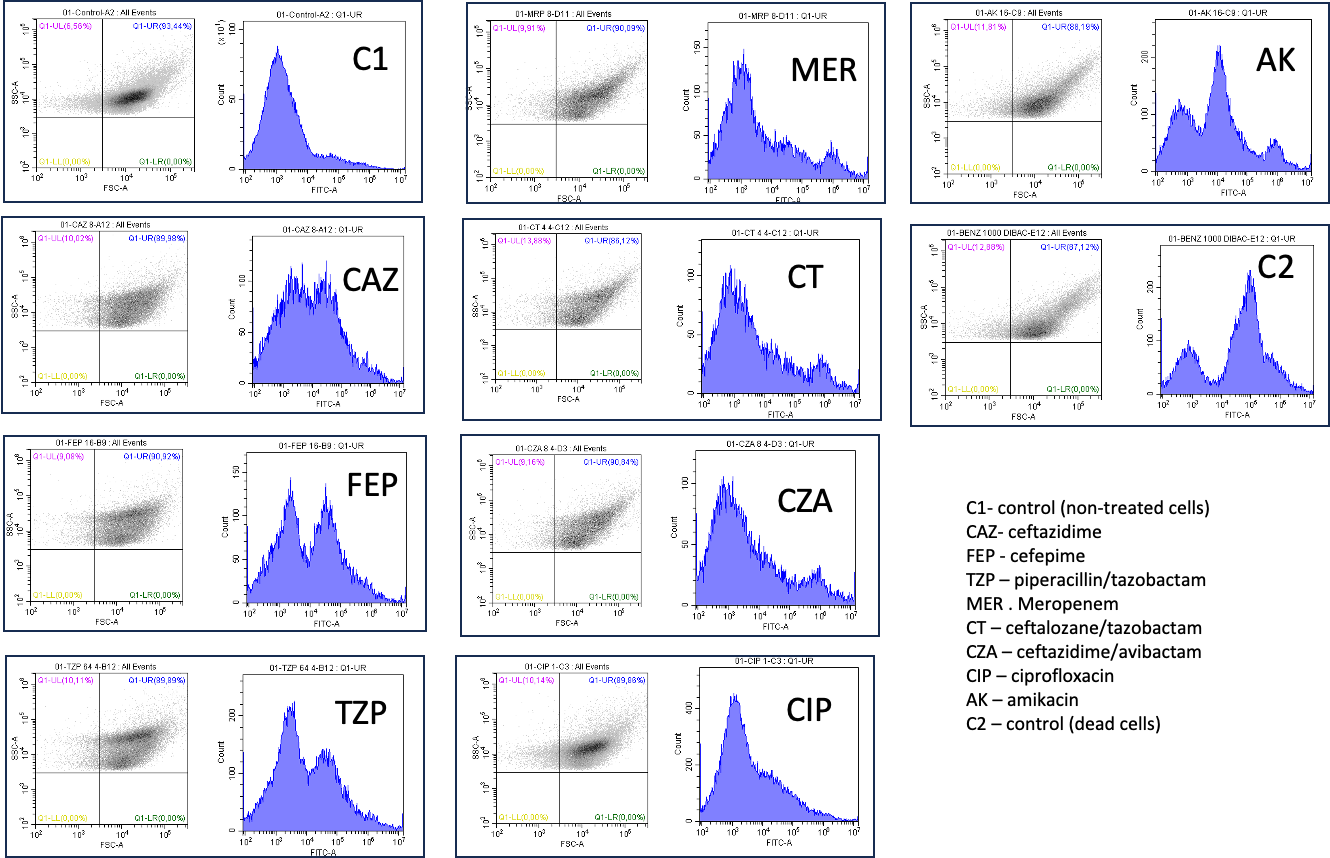


**Supplementary Figure 2.** Scattergram and histograms representing a fully susceptible *Pseudomonas aeruginosa* strain exposed for 1 hour to different antibiotics at breakpoint concentrations and a fluorescent dye; the control 1 (without antibiotic exposure) and control 2 (dead cells). SS-A side scatter, correlates with bacterial size; FSC-A forward scatter represents the complexity of bacteria. FITC-A means intensity of fluorescence in a log scale. Note the change on the scattergram and the Shift to the right of the intensity of fluorescence meaning susceptibility.
